# Crystallization Pathways of FABr-PbBr_2_-DMF and FABr-PbBr_2_-DMSO Systems: The Comprehensive Picture of Formamidinium-Based Low-Dimensional Perovskite-Related Phases and Intermediate Solvates

**DOI:** 10.3390/ijms232315344

**Published:** 2022-12-05

**Authors:** Sergey A. Fateev, Ekaterina I. Marchenko, Alexandra S. Shatilova, Victor N. Khrustalev, Eugene A. Goodilin, Alexey B. Tarasov

**Affiliations:** 1 Laboratory of New Materials for Solar Energetics, Department of Materials Science, Lomonosov Moscow State University, 1 Lenin Hills, 119991 Moscow, Russia; 2 Department of Geology, Lomonosov Moscow State University, 1 Lenin Hills, 119991 Moscow, Russia; 3 Inorganic Chemistry Department, Peoples’ Friendship University of Russia (RUDN University), 6 Miklukho-Maklay Str., 117198 Moscow, Russia; 4 N.D. Zelinsky Institute of Organic Chemistry RAS, 47 Leninsky Prosp., 119991 Moscow, Russia; 5 Department of Chemistry, Lomonosov Moscow State University, 1 Lenin Hills, 119991 Moscow, Russia

**Keywords:** hybrid perovskite, dimethylformamide solvent, dimethyl sulfoxide solvent, pathways of crystallization, intermediates

## Abstract

In this study, we systematically investigated the phase diversity and crystallization pathways of the FABr excessive regions of two ternary systems of FABr-PbBr_2_-DMF and FABr-PbBr_2_-DMSO (where FA^+^—formamidinium cations, DMF—dimethylformamide and DMSO—dimethyl sulfoxide solvents). In these systems, a new FA_3_PbBr_5_ phase with a structure containing chains of vertex-connected PbBr_6_ octahedra is discovered, and its crystal structure is refined. We experimentally assess fundamental information on differences in the mechanisms of crystallization process in FABr-PbBr_2_-DMF and FABr-PbBr_2_-DMSO systems and determine possible pathways of crystallization of hybrid perovskites. We show that intermediate solvate phases are not observed in the system with DMF solvent, while a number of crystalline solvates tend to form in the system with DMSO at various amounts of FABr excess.

## 1. Introduction

Organo-inorganic lead halide compounds with a perovskite structure of the general formula ABX_3_ [A = CH_3_NH_3_^+^ (MA^+^), CH(NH_2_)_2_^+^ (FA^+^); B = Pb^2+^, Sn^2+^; X = I^–^, Br^–^] as well as their low-dimensional derivatives have recently emerged as a promising new class of materials for solar cells and next-generation light-emitting diodes (LEDs) due to its broadly tunable photoluminescence (PL) (410–700 nm), high PL quantum yield (QYs = 50–90%), small full width at half maximum of PL peaks [1,2,3,4,5,6,7,8] and advantages of solution-processing techniques compatible to organic LEDs [9]. In particular, lead bromide materials with perovskite and perovskite-derived structures with bright-green PL emission might act as green emitters excited by standard blue-emitting diodes [10,11,12,13]. Solution methods of crystallization are popular for fabrication of bromide perovskite thin films with a good quality for light-emitting diodes with external quantum efficiency exceeding 20% [14,15]. The recent studies of the solution-processed crystallization of hybrid perovskites confirmed the crucial role of processing solvents and intermediate phases on the properties of final materials [16,17,18]. Additionally, it was previously identified that in systems with iodide anions and methylammonium cations, the crystallization of perovskite from DMF and DMSO solutions precedes the crystallization of intermediate solvate phases such as (MA)_2_Pb_3_I_8_·2Solv, (MA)_3_PbI_5_·Solv (Solv = DMF or DMSO) and (MA)_2_Pb_2_I_6_·2DMF, depending of the ratio of precursors [16,17], while in systems with bromide anions ions, it was found that crystallization proceeds without the formation of intermediate phases [19].

Unlike methylammonium and cesium the flat FA^+^ cation is considered to form plenty of layered two-dimensional (2D) polymorphs in the presence of organic halide excess [19,20] which can form multiphase heterostructures with FAPbBr_3_ 3D perovskite with highly tunable and improved optical properties such as enhanced EQE and reversed photochromism [21,22,23] of hybrid materials since it can form phases with different inorganic substructure dimensions (3D and 2D) [19,20]. Additionally, two adducts of formamidinium bromoplumbates with DMSO were recently discovered (FA_2_PbBr_4_·DMSO and FAPbBr_3_·DMSO) [17,24]. Thus, among the 3D hybrid perovskites FABr-PbBr_2_ system in the FABr excessive region is expected to have the most complex chemical equilibria during crystallization from widely used DMF and DMSO solvents because of competition between multiple possible perovskite-like phases and solvates. Understanding of these equilibria is essential for rational chemical engineering of solution-processed FA_x_PbBr_3+x_ films and devises. In the present work, we have investigated the phase diversity of the FABr-PbBr_2_-DMF and FABr-PbBr_2_-DMSO solution system for detailed optimization of perovskite film processing.

## 2. Results and Discussion

### 2.1. FABr-PbBr_2_-DMF/DMSO Systems: Equilibrium State

**Figure 1:**
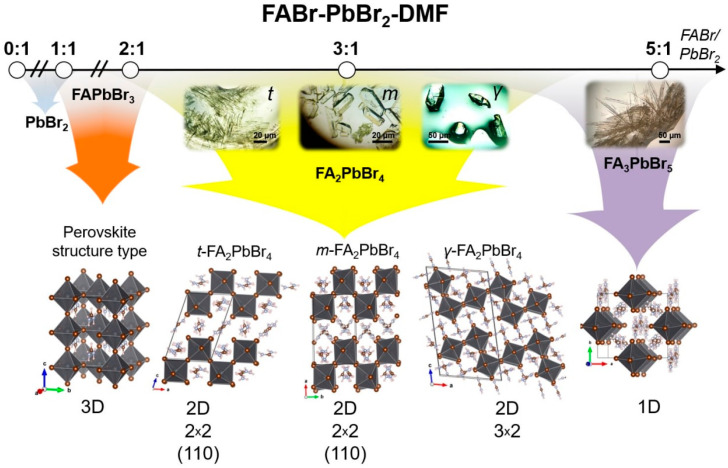
Phase diversity scheme of FABr-PbBr_2_-DMF system with different FABr:PbBr_2_ ratios. The approximate regions of the phase coexistence fields are shown by overlapping colored arrows.

To identify the equilibrium crystal phases in the FABr-PbBr_2_-DMF/DMSO systems, we observed the process of crystallization by drying drops of solutions of different compositions followed by isolation of the crystals to resolve the crystal structures by single-crystal X-ray diffraction methods. At room temperature (RT), the ternary solution system FABr-PbBr_2_-DMF in equilibrium state reveals five independent phases at different FABr/PbBr_2_ ratio (*r*) from 1 to 5 (Figure 1): FAPbBr_3_ perovskite, three different polytypes of two-dimensional (110) perovskite-derived structures FA_2_PbBr_4_ and FA_3_PbBr_5_ phase with one-dimensional perovskite-derived structure which is confirmed by XRD measurements. For FABr-PbBr_2_-DMSO system at RT conditions the crystallization process of 3D and 2D perovskites is accompanied by the appearance of intermediate solvate phases with FAPbBr_3_∙DMSO and FA_2_PbBr_4_∙DMSO compositions (Figure 1).

According to the XRD measurements for FABr-PbBr_2_-DMF system in the FABr/PbBr_2_ ratio (*r*) from 0 to 0.9 only PbBr_2_ phase is crystallized. At *r =* 0.9 ÷ 1, the PbBr_2_ coexist with the FAPbBr_3_ perovskite phase. Crystallization of phase-pure 3D perovskite FAPbBr_3_ was observed for the FABr/PbBr_2_ stoichiometric concentration and FABr-excessive compositions with *r =* 1.1 ÷ 1.5. An excess of FABr greater than 1.5 results in that the 3D perovskite phase crystallizes first, and crystallization of low-dimensional 2D perovskite FA_2_PbBr_4_ phases is observed from the remaining solution. Up to 4 different layered perovskite phases crystallizes directly from mother solution starting from *r* = 2.1. There are three layered perovskite-derived phases with the FA_2_PbBr_4_ stoichiometry corresponding to different crystal structures. The crystal structures of these polymorphs have already been published earlier [19,20]. The *t*-FA_2_PbBr_4_ and *m*-FA_2_PbBr_4_ compounds crystallize from DMF solution [19] while *γ*-FA_2_PbBr_4_ forms under distinctly different synthetic conditions precipitating under cooling from concentrated aqueous HBr [20]. Two of the FA_2_PbBr_4_ polymorphs have the crystal structure of (110)-oriented layered perovskite with ‘2 × 2’ arrangement of the inorganic layers whereas the third polymorph *γ*-FA_2_PbBr_4_ adopts a ‘3 × 2’ arrangement of the inorganic layers (see Figure 1). All structures consist of corner-shared ${\left[PbBr_{6}\right]}_{\infty }^{2-}$ octahedra forming corrugated layers separated with formamidinium interlayer cations. The main difference between first two polymorphs is the different Layer Shift Factor [25] (different stacking of adjacent inorganic layers). The m-FA_2_PbBr_4_ has a staggered [26] stacking of layers with LSF (0.5, 0.06), while the t-FA_2_PbBr_4_ represents the eclipsed stacking of the inorganic layers with LSF (0.06, 0.09). As described recently [19], growth of thin lamellar crystals of the *t*-FA_2_PbBr_4_ polymorph begins first, which is probably due to the heterogeneous nature of crystallization, then homogeneous crystallization of the *m*-FA_2_PbBr_4_ polymorph starts in the bulk of the solution usually accompanied by the dissolution and recrystallization of the *t*-FA_2_PbBr_4_ indicative of its metastable nature. The *γ*-FA_2_PbBr_4_ polymorph also crystallizes homogeneously at higher FABr-excess (*r* ≥ 3).

**Figure 2:**
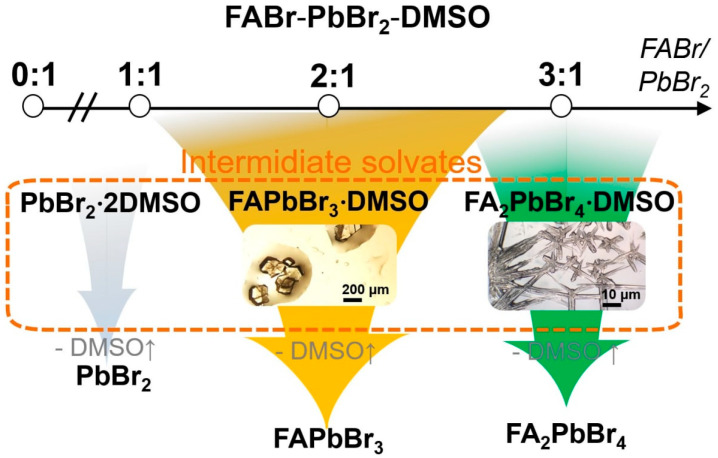
Phase diversity scheme of and FABr-PbBr_2_-DMSO system with different FABr:PbBr_2_ ratios. The approximate regions of the phase coexistence fields are shown by overlapping colored arrows.

In contrast, in the FABr-PbBr_2_-DMSO system, only solvate phases crystalize firstly from solutions for all FABr:PbBr_2_ ratios (Figure 2). For *r* in the range from 0 to 0.9, crystals of the known phase PbBr_2_∙2DMSO [27] grow from the solution. Precipitation of another recently discovered solvate phase, FAPbBr_3_∙DMSO [24], is observed from solutions with a stoichiometric FABr:PbBr_2_ ratio and with up to two-fold an excess of FABr. Crystallization of the DMSO solutions with r > 2 results in the formation of another adduct with a chain structure of corner-shared ${\left[Pb\left(DMSO\right)Br_{5}\right]}_{\infty }^{3-}$ octahedra [18]. Interestingly, that structures of all tree DMSO-solvates are characterized by direct coordination of Pb^2+^ ions with DMSO molecules via donor-acceptor Pb∙∙∙O bonds as quite typical for lead dihalides, but rare for the case of ternary (hybrid) haloplumbates [17]. This structural difference is caused by the high donor ability of DMSO models (DN—donor number—is comparable to the bromide anion) [17], that explains the relatively high thermodynamic stability of these solvates because they do not decompose in dry air even after the solution has been completely dried.

**Figure 3:**
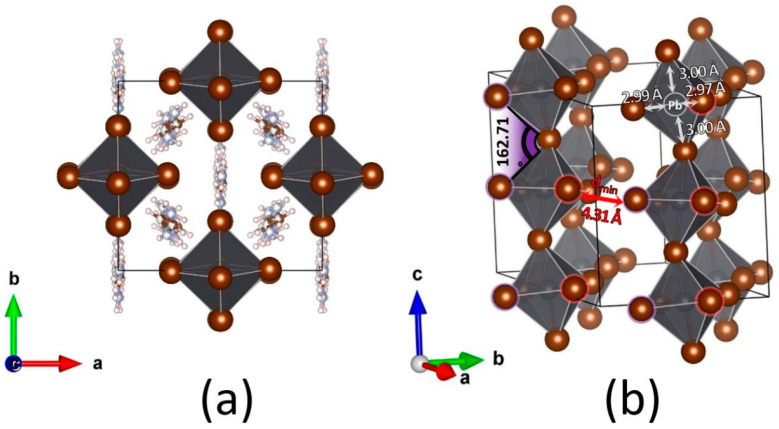
The crystal structure of a new 1D FA_3_PbBr_5_ phase in different projections: xy (**a**) and oxonometric (**b**). The vertex-connected chains of PbBr_6_ octahedra shown in polyhedral representation. The formamidinium cations was removed for clarity (**b**).

Surprisingly, a new one-dimensional structure of the FA_3_PbBr_5_ composition with corner-shared chains of ${\left[PbBr_{6}\right]}_{\infty }^{3-}$ octahedra is observed (Figure 3) in the FABr-PbBr_2_-DMF system at *r* ≥ 4. The crystal structure of this phase was refined from a single crystal X-ray diffraction experiment. Crystallographic and refinement data for this crystal structure are shown in Table 1 and Crystallographic Information File (CIF) in SI. The FA_3_PbBr_5_ phase crystallizes in the monoclinic space group *C*2/*c* with the unit cell parameters *a* = 12.466(3) Å, *b* = 11.465(2) Å, *c* = 11.868(3) Å and the monoclinic angle β equal to 99.029(6)°. The number of formula units z = 4. Pb-Br bond lengths in PbBr_6_ octahedra are 2.97–3.00 Å. The octahedra in the chains are tilted relative to each other so that the Pb-Br-Pb angles are 162.71°. The shortest Br∙∙∙Br distance between neighboring chains of octahedra is 4.31 Å (Figure 3b). This distance between adjacent chains of octahedra is small and is approximately equal to the diagonal of PbBr_6_ octahedron. Therefore, the small band gap can be expected due to the partial interchain overlapping of bromine *p*-orbitals.

**Figure 4:**
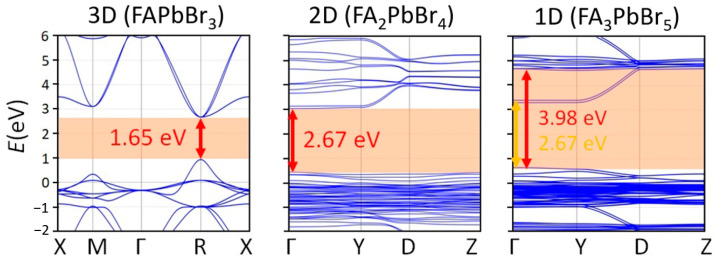
Calculated band structures for FAPbBr_3_ (3D), FA_2_PbBr_4_ (2D) and FA_3_PbBr_5_ (1D) phases.

To compare the electronic properties of the observed 3D, 2D, 1D hybrid perovskite and low-dimensional perovskite-related phases with different FABr:PbBr_2_ ratios, we calculated the band structures of these compounds using the density functional theory (DFT) methods. The obtained results are consistent with the concept of quantum confinement for hybrid perovskite and perovskite-derived semiconductors [28]: the 3D FAPbBr_3_ perovskite is a direct-gap semiconductor with a minimal band gap of 1.65 eV at R point of the Brillouin zone; the band gap increases and the band dispersion becomes smaller (Figure 4) with a decrease in the dimension of the inorganic substructure. Particularly, for 2D FA_2_PbBr_4_ perovskite, the band gap at the Γ point of the Brillouin zone is 2.67 eV. The 1D FA_3_PbBr_5_ phase also has a direct band gap of 2.67 eV equal to the 2D phase, but in contrast to FA_2_PbBr_4_, the band structure of 1D phase is characterized by separated lower and higher conduction bands: the first has density of states much lower than for the 2D structure, while the second has comparable density of states. Such distinct separation of conduction band could be associated with the small distance between the chains neighboring chains of corer-shared octahedra (and, consequently, the overlapping of the halogen orbitals) [29].

### 2.2. FABr-PbBr_2_-DMF/DMSO System: Thin Films

**Figure 5:**
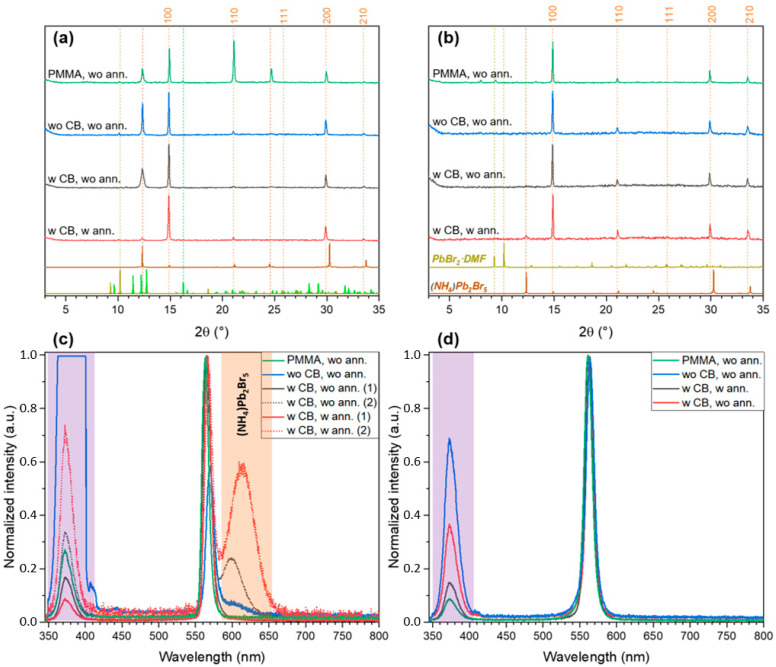
The XRD patterns (**a**,**b**) and PL spectra (**c**,**d**) of thin films with FABr:PbBr_2_ = 1 spin-coated at different conditions from DMSO (**a**,**c**) and DMF (**b**,**d**) solutions. The dashed orange lines denote the reflections of FAPbBr_3_ perovskite, other dashed lines indicate the positions of the most relevant reflections corresponding to reference XRD pattens plotted below ((NH_4_)Pb_2_Br_5_ by brown, PbBr_2_·DMF by olive, FAPbBr_3_·DMSO by lime green). The light violet strip corresponds to the main band in the spectra of UV lamp (excitation source), the orange band corresponds to PL band of (NH_4_)Pb_2_Br_5_.

Further we investigated the influence of spin-coating conditions (type of solvent and presence of antisolvent and annealing steps) on the phase composition of thin films. In the particular case of the stoichiometric solution (FABr:PbBr_2_ = 1:1) in DMF, crystallization begins with the formation of PbBr_2_∙DMF adduct detected for intermediate films captured under a thick PMMA layer (Figure 5b). We also observed a small reflection of unknown phase at 10.1 2θ degree (Figure 5a) for the films spin-coated without antisolvent and without annealing from DMSO solution. This reflection may be associated with a new PbBr_2_∙DMSO phase isostructural with the known PbBr_2_∙DMF adduct. Interestingly, the crystallization of perovskite from the DMSO solution is accompanied with precipitation of the impurity phase (NH_4_)Pb_2_Br_5_ [30] (Figure 5a) associated with the partial decomposition of formamidinium cations in solution in accordance with the reaction HC(NH_2_)_2_ = NH_4_ + HCN↑. The formation of this impurity was also observed in earlier works in the case of nanocrystals [10], thin films, as well as single crystals [31]. The decomposition reaction apparently proceeds much more actively in DMSO solutions as confirmed by a larger proportion of the impurity phase both in the initial films and in the annealed samples according to XRD (Figure 5a). This can be explained by the higher basicity of DMSO, which leads to a more efficient deprotonation of the formamidinium cation, as well as a faster decomposition of the resulting formamidine [32].

The presence of (NH_4_)Pb_2_Br_5_ phase is also corroborated by the low-temperature PL spectra revealing the broad peak with maxima about 600–620 nm which is close to PL maximum of isostructural KPb_2_Br_5_ phase (620–650 nm) [33]. It should be noted that the impurity phase and 3D perovskite can be distributed in films rather unevenly, which is expressed in a different ratio of the PL intensities of these phases at different points of the samples (Figure 5c). In the case of DMF solvent, the amount of (NH_4_)Pb_2_Br_5_ impurity phase is much lower (observed only for annealed film), but no traces of this phase is observed in PL spectra (Figure 5d).

**Figure 6:**
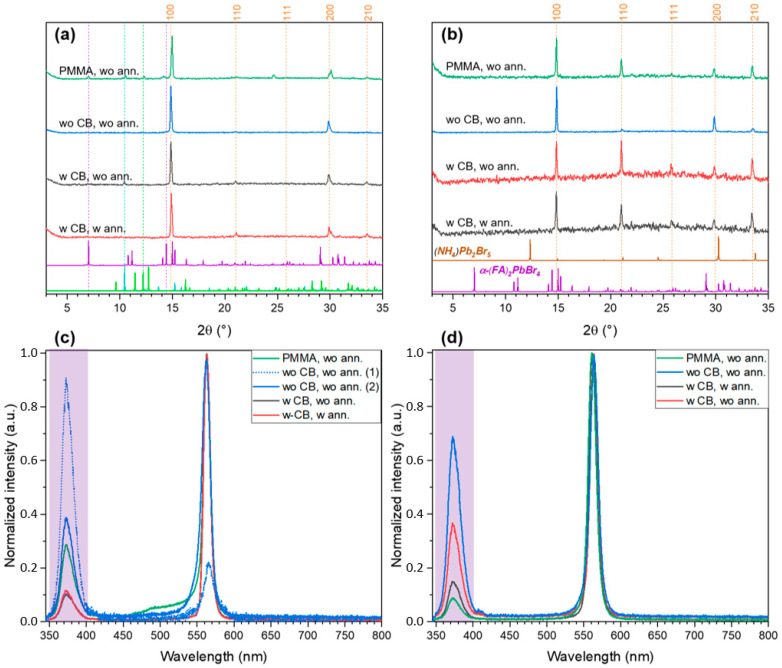
The XRD patterns (**a**,**b**) and PL spectra (**c**,**d**) of thin films with FABr:PbBr_2_ = 1.5 spin-coated at different conditions from DMSO (**a**,**c**) and DMF (**b**,**d**) solutions. The dashed orange lines denote the reflections of FAPbBr_3_ perovskite, other dashed lines indicate the positions of the most relevant reflections corresponding to reference XRD pattens plotted below (FA_2_PbBr_4_ by vivid violet, FAPbBr_3_·DMSO by lime green, FA_2_PbBr_4_·DMSO by turquoise). The light violet strip corresponds to the main band in the spectra of UV lamp (excitation source).

Increasing the FABr:PbBr_2_ ratio up to 1.5:1 makes it possible to obtain single-phase FAPbBr_3_ perovskite films. In the case of DMF, the perovskite crystallizes directly from solution (Figure 6b), while for DMSO, crystallization proceeds through the formation of solvates FAPbBr_3_·DMSO and FA_2_PbBr_4_·DMSO (Figure 6a) and intermediate formation of *t*-FA_2_PbBr_4_ phase, the gradual decomposition of which provides higher crystallinity and better optical properties of the final films (compare PL intensity for Figures S7 and S9). For both DMSO and DMF, the acceleration of crystallization due to the addition of an antisolvent naturally leads to a decrease in crystallinity; while, in the case of DMSO, annealing promotes an additional recrystallization due to the presence of residual solvent in deposited films.

**Figure 7:**
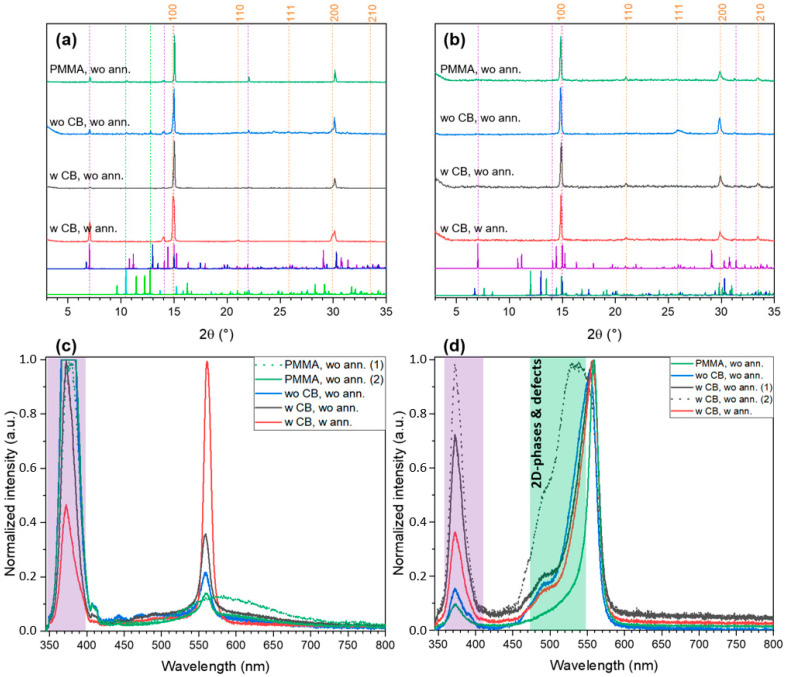
The XRD patterns (**a**,**b**) and PL spectra (**c**,**d**) of thin films with FABr:PbBr_2_ 2:1 spin-coated at different conditions from DMSO (**a**,**c**) and DMF (**b**,**d**) solutions. The dashed orange lines denote the reflections of FAPbBr_3_ perovskite, other dashed lines indicate the positions of the most relevant reflections corresponding to reference XRD pattens plotted below. (*t*-FA_2_PbBr_4_ by vivid violet, *m*-FA_2_PbBr_4_ by dark blue, FAPbBr_3_·DMSO by lime green, FA_2_PbBr_4_·DMSO by turquoise).

For the FABr:PbBr_2_ ratio of 2:1, crystallization from DMSO also begins with solvates FAPbBr_3_·DMSO and FA_2_PbBr_4_·DMSO (Figure 7a, unannealed films) and then they decompose gradually to form 3D perovskites nuclei surrounded by the matrix of 2D phase (Figure 7a, annealed film). Notably, only the *t*-FA_2_PbBr_4_ phase is formed probably due to faster heterogenic crystallization. Crystallization from DMF is faster due to higher volatility of solvent and lover solvating energy, which results in lower crystallinity of the films (lower integral intensity, Figure 7b) and higher density of stacking faults (absence of small-angle layer-planes-related reflections). The PL data further corroborates the conclusion from powder the XRD results. All films spin-coated from DMSO solutions demonstrate intense narrow and slightly blue-shifted PL peak of 3D perovskite FAPbBr_3_ (559–562 nm, Figure S1) with a shoulder corresponding to broad PL maximum ofFAPbBr_3_·DMSO solvate (in the case of PMMA-capped intermediate film only broad PL of solvate is observed in some pints of the film). Whereas DMF-processed films show higher blue shift of the PL band (553–562 nm, Figure S1) of 3D perovskite along with broad shoulder associated with radiative recombination caused by stacking faults and other 2D-defects [34] characteristic for FA_2_PbBr_4_ phase [19] and other layered perovskites with small intralayer cations [35,36,37].

**Figure 8:**
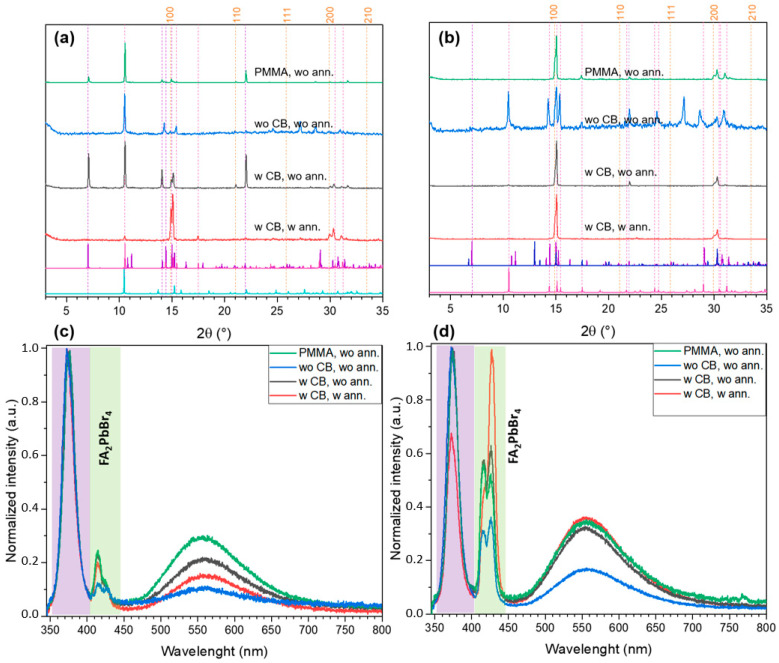
The XRD patterns (**a**,**b**) and PL spectra (**c**,**d**) of thin films with FABr:PbBr_2_ 3:1 spin-coated at different conditions from DMSO (**a**,**c**) and DMF (**b**,**d**) solutions. The dashed orange lines denote the reflections of FAPbBr_3_ perovskite, other dashed lines indicate the positions of the most relevant reflections corresponding to reference XRD pattens plotted below (FA_3_PbBr_5_ by magenta, *t*-FA_2_PbBr_4_ by vivid violet, FA_2_PbBr_4_·DMSO by turquoise, *m*-FA_2_PbBr_4_ by dark blue).

For the highest used FABr:PbBr_2_ ratio (*r* = 3), crystallization from DMSO starts with intermediate formation of the *t*-FA_2_PbBr_4_ layered phase which recrystallizes and reacts with FABr excess under annealing to form chained 1D FA_3_PbBr_5_ phase (Figure 8a). In the case of DMF, the FA_3_PbBr_5_ crystallizes directly from the solution (no intermediate phases were identified according XRD, Figure 8b). Using of antisolvent leads to texturing of the films along (002) and (004) planes resulting in XRD pattern asimilar to 3D perovskite (Figure 8b) while it is not present in the films with r = 3:1 because of their white color and the absence of FAPbBr_3_-related photoluminescence (Figure 8c,d). Without antisolvent, the reflections of the low-dimensional phases manifest themselves, but integral intensity becomes much lower. The PL spectra are consistent with the XRD data: the impurities of 2D perovskite *t*-FA_2_PbBr_4_ reveal itself by characteristic doubled PL maximum at 415–430 nm, whereas FA_3_PbBr_5_ 1D phase is likely characterized by broad asymmetric PL peak with maximum at 560 nm, which is probably associated with radiative recombination via self-trapped excitons (Figure 8c,d).

In conclusion, we briefly discuss the effect of stoichiometry and processing conditions on the photoluminescent characteristics (peak position and intensity) of thin films at room temperature. In contrast to PL at liquid nitrogen temperature, measurements at room temperature were carried out with precise focusing of the laser beam (405 nm) on the sample surface and with normalization relative to the standard, which makes it possible to quantitatively compare the intensity for different samples. The position of the PL peak for films with r = 1 at room temperature corresponds to the standard peak position for 3D perovskite (~555 nm) (Figures S2–S5). An increase in r to 1.5 leads to a shift in the PL peak to a region below 550 nm (Figures S6 and S8), which probably reflects a smaller crystallite size or a higher stacking error concentration in 3D perovskite. Along with the shift of the peak, the average PL intensity also increases with increasing r for most deposition conditions, both in the case of DMSO and DMF (Figures S7 and S9). A further increase in r to 2 has the opposite effect on the PL intensity in two solutions: in the case of DMSO, the intensity drops sharply (Figures S10 and S11), which is apparently associated with a significant decrease in the proportion of 3D perovskite, while in the case of DMF, the intensity, on the contrary, reaches a maximum (Figures S12 and S13) due to the formation of the FAPbBr_3_@FA_2_PbBr_4_ composite with efficient charge transfer. In the case of the maximum ratio r = 3, the PL intensity in the region of 500–600 nm decreases many times (Figures S14–S17), and the peak becomes even more asymmetric, shifting to the blue region. Apparently, this broadened emission band corresponds to “3D-like” structural defects in the matrix of the low-dimensional chain phase, which, like the 2D phase, does not exhibit its own photoluminescence at room temperature.

## 3. Materials and Methods

### 3.1. Materials

Formamidinium bromide (CH(NH_2_)_2_Br = FABr, Dyesol, Queanbeyan, Australia), lead bromide (PbBr_2_, 99.99%, TCI, Tokyo, Japan), dimethylsulfoxide (DMSO, anhydrous, >99.99%, Sigma-Aldrich, St. Louis, MO, USA), dimethylformamide (DMF, anhydrous, >99.8%, Sigma-Aldrich, USA), chlorobenzene (>99.8%, Sigma-Aldrich, USA).

### 3.2. Single Crystal Growth

Experiments to isolate low dimensional formamidinium-containing phases were made using several solutions in DMF and DMSO with FABr/PbBr_2_ ratio from 0 to 5 for DMF and from 0 to 3 for DMSO. Then, we placed drops of all solutions on quartz glass and observed the process of crystal growth. Typical crystals from each droplet were isolated from the solution using nylon loop, blotted and immediately transferred for X-ray measurements and solved as described below.

### 3.3. Single-Crystal X-ray Diffraction (XRD)

The single-crystal X-ray diffraction data set were collected on Bruker D8 QUEST PHOTON-III CCD diffractometer (T = 100 K, λ(MoKα)-radiation, graphite monochromator, ω- and φ-scanning mode). The data were indexed and integrated using the SAINT program [38], and then scaled and corrected for absorption using the SADABS program [39]. The structure was solved by intrinsic phasing modification of direct methods, and refined by a full-matrix least-squares technique on *F^2^* with anisotropic displacement parameters for all nonhydrogen atoms. All calculations were carried out using the SHELXTL program suite [40].

The single-crystal X-ray diffraction study of FAPbBr_3_·DMSO was carried out on the ‘RSA’ beamline (T = 100 K, λ = 0.79313 Å) of the Kurchatov Synchrotron Radiation Source. In total, 720 frames were collected with an oscillation range of 1.0° in the φ scanning mode using two different orientations of the crystal. The semiempirical correction for absorption was applied using the Scala program [41]. The data were indexed and integrated using the utility iMOSFLM 7.2.2 from the CCP4 7.1 software suite [42,43].

### 3.4. Powder X-ray Diffraction

Powder XRD patterns were collected using a Bruker Advance D8 diffractometer (Germany) with Cu Kα irradiation in the Bragg−Brentano geometry. The patterns were recorded in a 3−35° 2θ range with a 0.02° step.

### 3.5. Thin Films Preparation

Glass substrates were cleaned with detergent, flushed with distilled water and then washed in ultrasonic bath in distilled water three times. Further the substrates were cleaned with UV-ozone for 15 min before use.

Solutions of FABr-PbBr_2_ in DMF and DMSO were prepared at room temperature under stirring during 16 h in argon filled glove box, unless otherwise indicated. The FABr:PbBr_2_ ratio (*r*) in solutions was variated from 1 to 3.1, the concentration of all solutions C[Pb^2+^] was 1.25 M. To reveal the influence of crystallization conditions on the phase composition thin films were fabricated at different modes (in all cases the perovskite solution (30 μL) was distributed evenly over the glass and then sample was rotated at 4000 rpm for 30 s): with antisolvent (chlorobenzene, further CB) or without antisolvent, as well as with polymethylmethacrylate solution in CB as antisolvent (concentration of PMMA in CB—75 mg/mL). The latter was used to isolate and preserve the intermediate phases crystallized initially in thin films prior to measurements. Half of the films obtained with antisolvent were annealed at 80 °C for 20 min. All thin films except ones fabricated without chlorobenzene were covered by PMMA layer spin-coated from solution of 75 mg/mL at 4000 rpm to prevent exposure to moisture during the measurements.

### 3.6. Photoluminescence Measurements

Steady-state PL measurements were performed on a microscope assembled using Thorlabs optomechanical components at the temperature of 77 K by immersing the samples in the liquid nitrogen. Samples were photoexcited by UV lamp with a main wavelength of 365 nm. The PL spectra were recorded using the Flame (Ocean Insight, Orlando, United States) spectrometer.

### 3.7. DFT Calculations

Electronic band structure calculations were obtained with the density functional theory (DFT) implemented with the Quantum ESPRESSO (version 6.1) [44,45]. The electronic exchange-correlations were treated by the Perdew–Burke–Ernzerhof (PBE) under a generalized gradient approximation (GGA) [46], and the OTFG Ultrasoft pseudo-potential was used to describe the interaction between electrons and ions [47]. The cut-off energy of plane wave of the system is set at 435 eV to ensure the convergence of energy and configuration of the system at the level of quasi-complete plane wave base. In the self-consistent field operation, Pulay density mixing method is adopted, and the self-consistent field is set as 1.0 × 10^−6^ eV/atom. The valence electrons involved in the calculation are Pb-6s^2^6p^2^ and Br-5s^2^5p^5^. The calculations did not include spin–orbit coupling. A visualization of crystal structures was performed using the VESTA program [48].

## 4. Conclusions

In this study, we systematically investigated the phase diversity and crystallization pathways in two ternary systems of FABr-PbBr_2_-DMF and FABr-PbBr_2_-DMSO. Based on investigation of the crystallization under equilibrium conditions, we identified in total 5 phases coexisting with solution for FABr-PbBr_2_-DMF system (FAPbBr_3_, *t*-FA_2_PbBr_4_, *m*-FA_2_PbBr_4_, *γ*-FA_2_PbBr_4_, FA_3_PbBr_5_) and 3 phases crystallizing from saturated DMSO solutions (PbBr_2_·DMSO, FAPbBr_3_·DMSO, FA_2_PbBr_4_·DMSO). The crystal structure of the newly discovered FA_3_PbBr_5_ phase contains chains of vertex-connected ${\left[PbBr_{6}\right]}_{\infty }^{3-}$ octahedra separated by formamidinium with the shortest Br∙∙∙Br distance between neighboring chains of octahedra of 4.31 Å.

DMF and DMSO solvents exert a decisive effect on the crystallization process and phase composition of solution-processed thin films. Crystallization from a stoichiometric solution (FABr:PbBr_2_ = 1:1) is accompanied by the formation of a impurity phase (NH_4_)Pb_2_Br_5_ associated with the partial decomposition of FA^+^ cations in solution, which proceeds much more actively in the DMSO solution. Increasing the FABr:PbBr_2_ ratio up to 1.5:1 makes it possible to obtain single-phase FAPbBr_3_ perovskite films. In the case of DMF, the perovskite crystallizes directly from the solution, while for DMSO, crystallization proceeds through the formation of intermediate solvates (FAPbBr_3_·DMSO and FA_2_PbBr_4_·DMSO), the gradual decomposition of which provides higher crystallinity and better optical properties of the final film. For the ratios of 2:1 and 3:1, crystallization from solutions in DMSO also starts with the solvates which are decomposed with initial formation of higher-dimensional phases (3D in the case of 2:1 and 2D for 3:1) followed by crystallization of the bulk low-dimensional wide-bandgap phases. In the case of DMF, the FA_2_PbBr_4_ and FA_3_PbBr_5_ crystallizes directly from solution (no intermediate phases were detected). Faster crystallization results in lower crystallinity and presumably higher density of stacking faults in low-dimensional phases leading to formation of additional PL-emissive centra in corresponding 2D and 1D phases.

Associated Content: The Supporting Information file contains the raw steady-state photoluminescence (PL) spectra measured at standard conditions for the thin film samples prepared at different stoichiometries, solvents, and processing conditions, as well as the diagrams reflecting the dependences of PL parameters (intensity and position of the maximum) on the and processing conditions.

## Figures and Tables

**Table 1:** Crystal and refinement data for new FA_3_PbBr_5_ phase.

Phase	FA_3_PbBr_5_
Appearance	needle-shaped crystals
Crystal system	monoclinic
Space group	*C*2/*c*
Unit cell parameters	*a* = 12.466(3) Å, *b* = 11.465(2) Å, *c* = 11.868(3) Å, α = 90°, β = 99.029(6)°, γ = 90°
Cell volume, Å^3^	1675.2(7)
Z	4
Density (calculated), g/cm^3^	2.943
Reflections collected	12281
Independent reflections	3040
data/restraints/parameters	3040/41/72
goodness-of-fit	1.013
final *R* indices [*I* > 2σ(I)]	*R*_obs_ = 0.0571, w*R*_obs_ = 0.1347
*R* indices [all data]	*R*_all_ = 0.0931, w*R*_all_ = 0.1553

*R* = ∑||F_o_| − |F_c_||/∑|F_o_|, w*R* = {∑[w(|F_o_|^2^ − |F_c_|^2^)^2^]/∑[w(|F_o_|^4^)]}^1/2^.

## Data Availability

The data that support the plots within this paper are available from the corresponding author upon request.

## Supplementary Materials

The following supporting information can be downloaded at: https://www.mdpi.com/article/10.3390/ijms232315344/s1.
