# Supplementary material for: Crystallization Pathways of FABr-PbBr2-DMF and FABr-PbBr2-DMSO Systems: The Comprehensive Picture of Formamidinium-Based Low-Dimensional Perovskite-Related Phases and Intermediate Solvates
Source: Int J Mol Sci. 2022 Dec 5;23(23):15344. doi: 10.3390/ijms232315344 (PMC9739158; doi:10.3390/ijms232315344)
Supplement: Supplementary file 1 [file ijms-23-15344-s001.zip › Figures S1-S17.pdf]

SUPPORTING INFORMATION
for the article

“Crystallization pathways of FABr-PbBr$_2$-DMF and FABr-PbBr$_2$-DMSO systems: the comprehensive picture of formamidinium-based low-dimensional perovskite-related phases and intermediate solvates”

Sergey A. Fateev [a], Ekaterina I. Marchenko [a,b], Alexandra S. Shatilova [a], Victor N. Khrustalev [c, d], Eugene A. Goodilin[a,e] and Alexey B. Tarasov*[a,e]

[a] *Laboratory of New Materials for Solar Energetics, Department of Materials Science, Lomonosov Moscow State University; 1 Lenin Hills, 119991, Moscow, Russia;*
[b] *Department of Geology, Lomonosov Moscow State University; 1 Lenin Hills, 119991, Moscow, Russia;*
[c] *Inorganic Chemistry Department, Peoples’ Friendship University of Russia (RUDN University), 6 Miklukho-Maklay Str., 117198, Moscow, Russia*
[d] *N.D. Zelinsky Institute of Organic Chemistry RAS, 47 Leninsky Prosp., 119991, Moscow, Russia*
[e] *Department of Chemistry, Lomonosov Moscow State University; 1 Lenin Hills, 119991, Moscow, Russia*
**corresponding author*
e-mail: alexey.bor.tarasov@yandex.ru

The Supporting Information file contains the raw steady-state photoluminescence (PL) spectra measured at standard conditions for the thin film samples prepared at different stoichiometries, solvents, and processing conditions, as well as the diagrams reflecting the dependences of PL parameters (intensity and position of the maximum) on the and processing conditions.

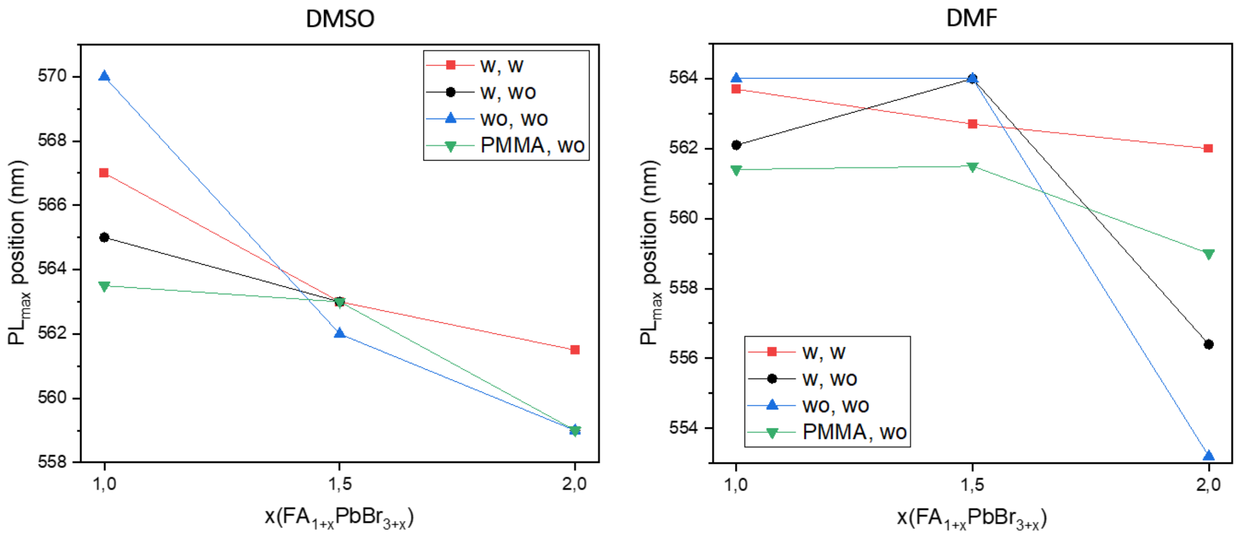

Figure S1. The position of PL maximum of 3D perovskite depending on FABr:$PbBr_2$ ratio.

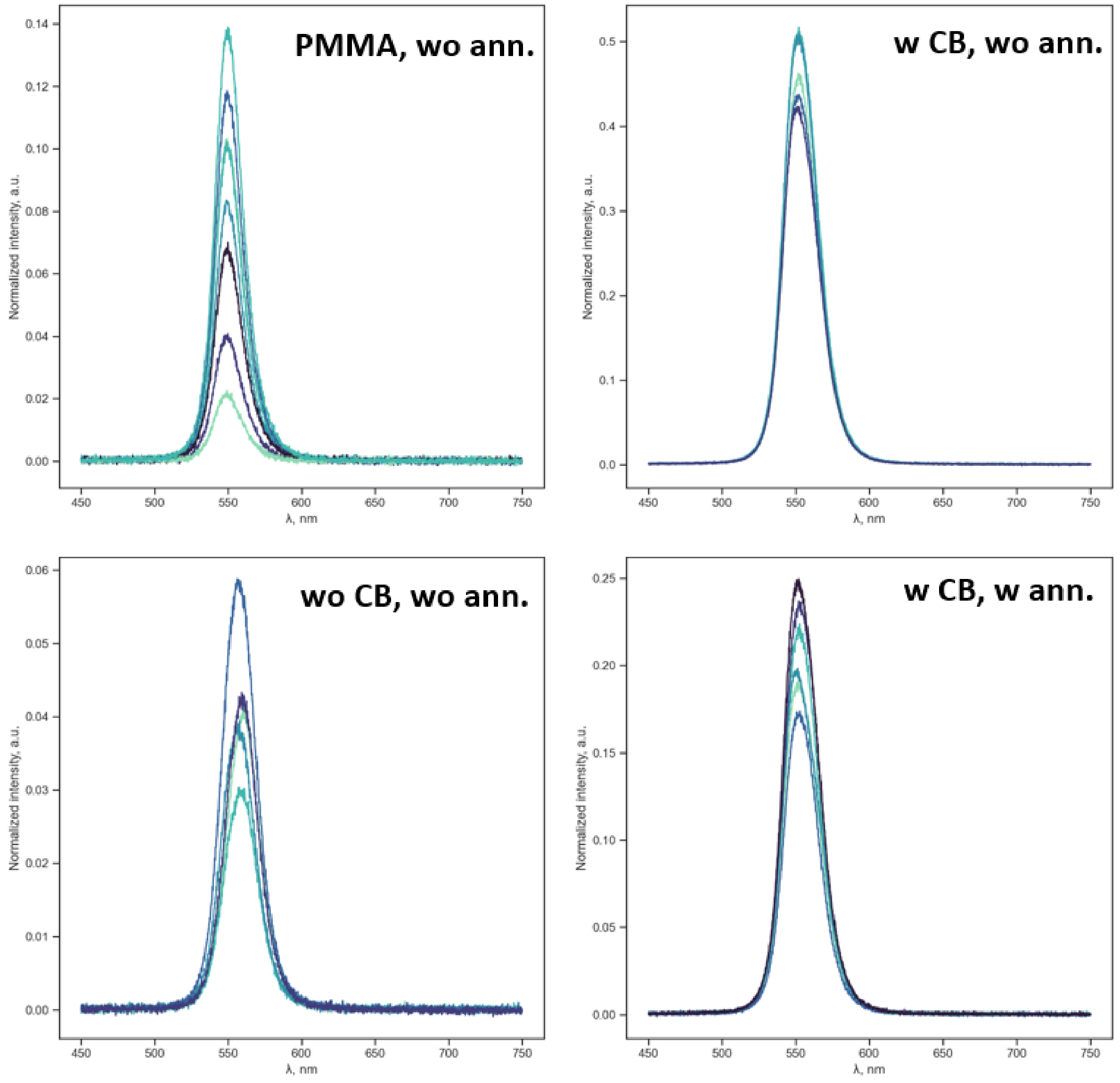

Figure S2. The PL spectra of the film spin-coated from DMF solution with $FABr:PbBr_2$ ratio of 1:1 for different processing conditions. Different spectra for each graph correspond to different points on the sample.

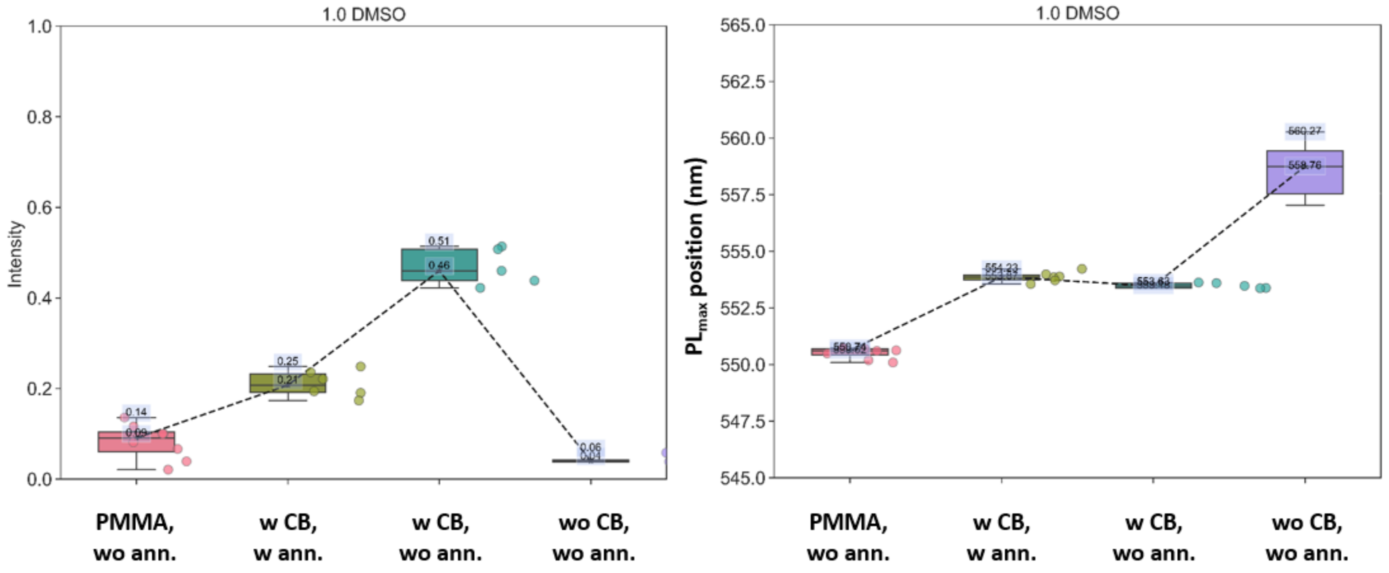

Figure S3. The PL intensity (relative to the standard) and position of PL maximum (nm) for different processing conditions (DMSO, $FABr:PbBr_2$ = 1:1). The statistical spread corresponds to the spectra above.

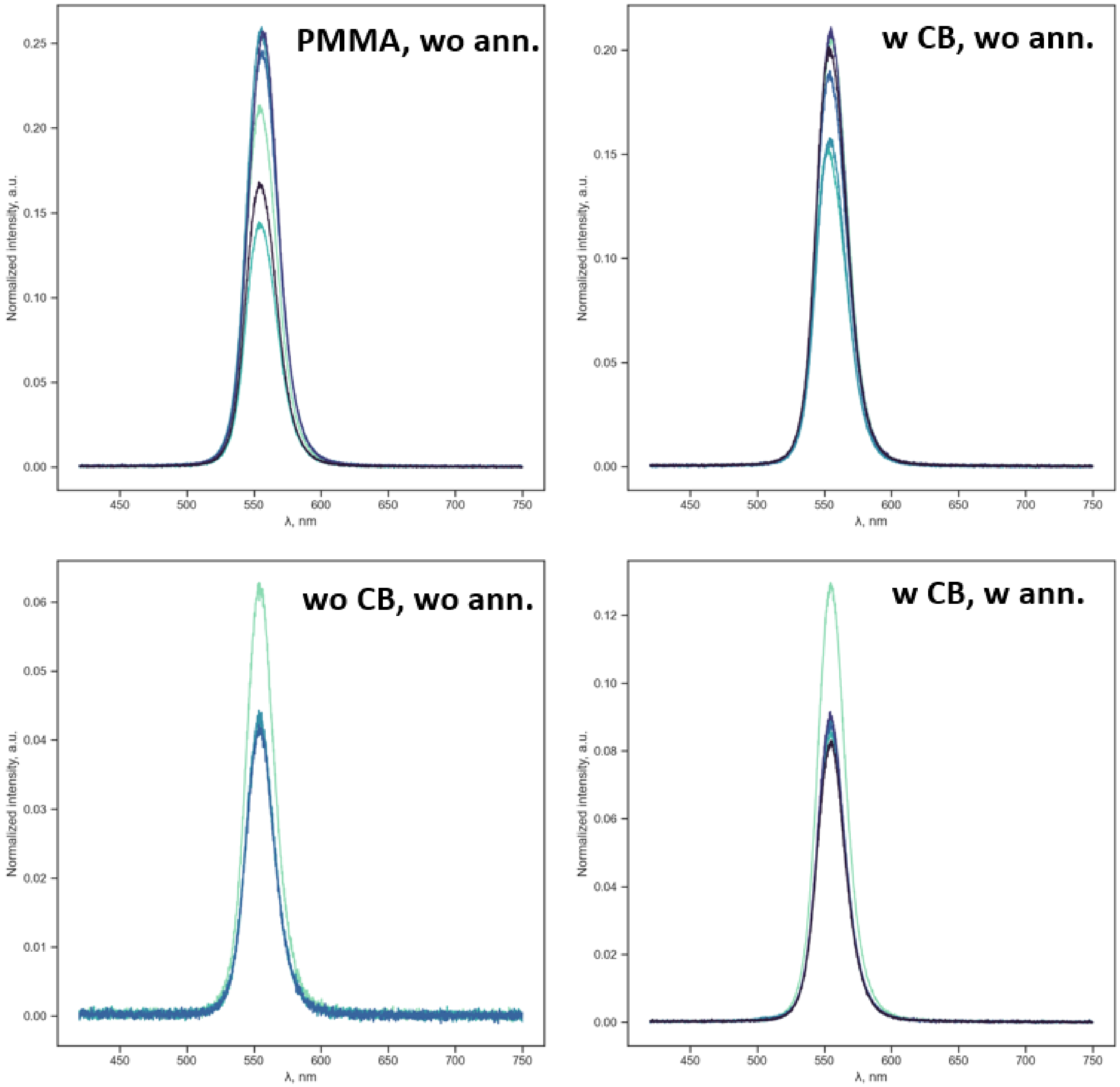

Figure S4. The PL spectra of the film spin-coated from DMF solution with $FABr:PbBr_2$ ratio of 1:1 for different processing conditions. Different spectra for each graph correspond to different points on the sample.

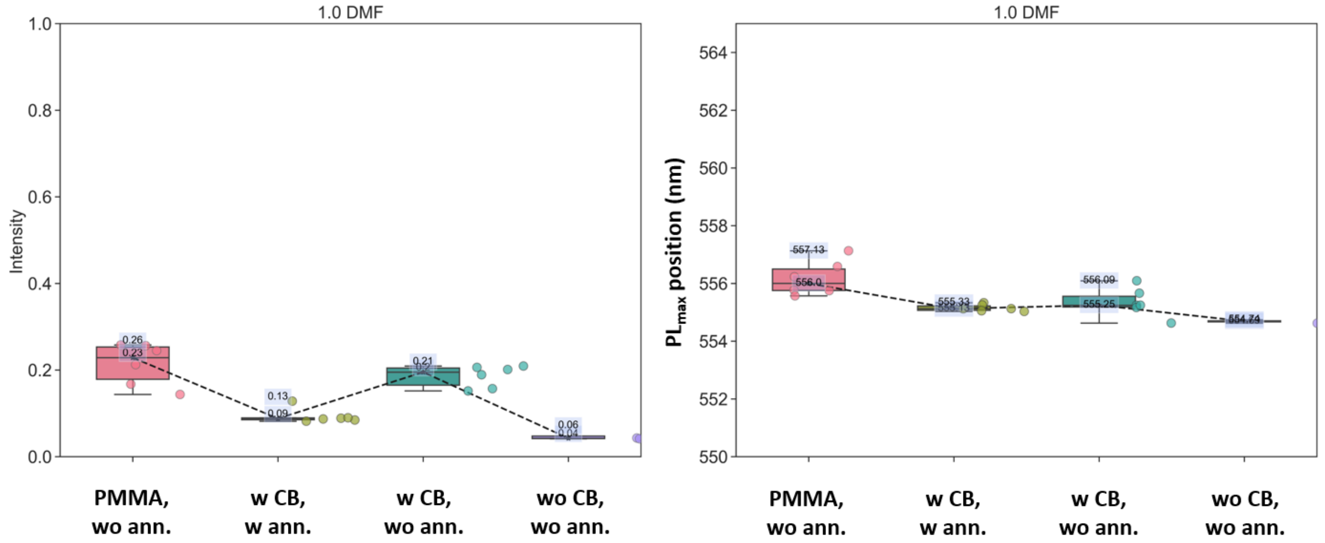

Figure S5. The PL intensity (relative to the standard) and position of PL maximum for different processing conditions (DMF, $FABr:PbBr_2$ = 1:1). The statistical spread corresponds to the spectra above.

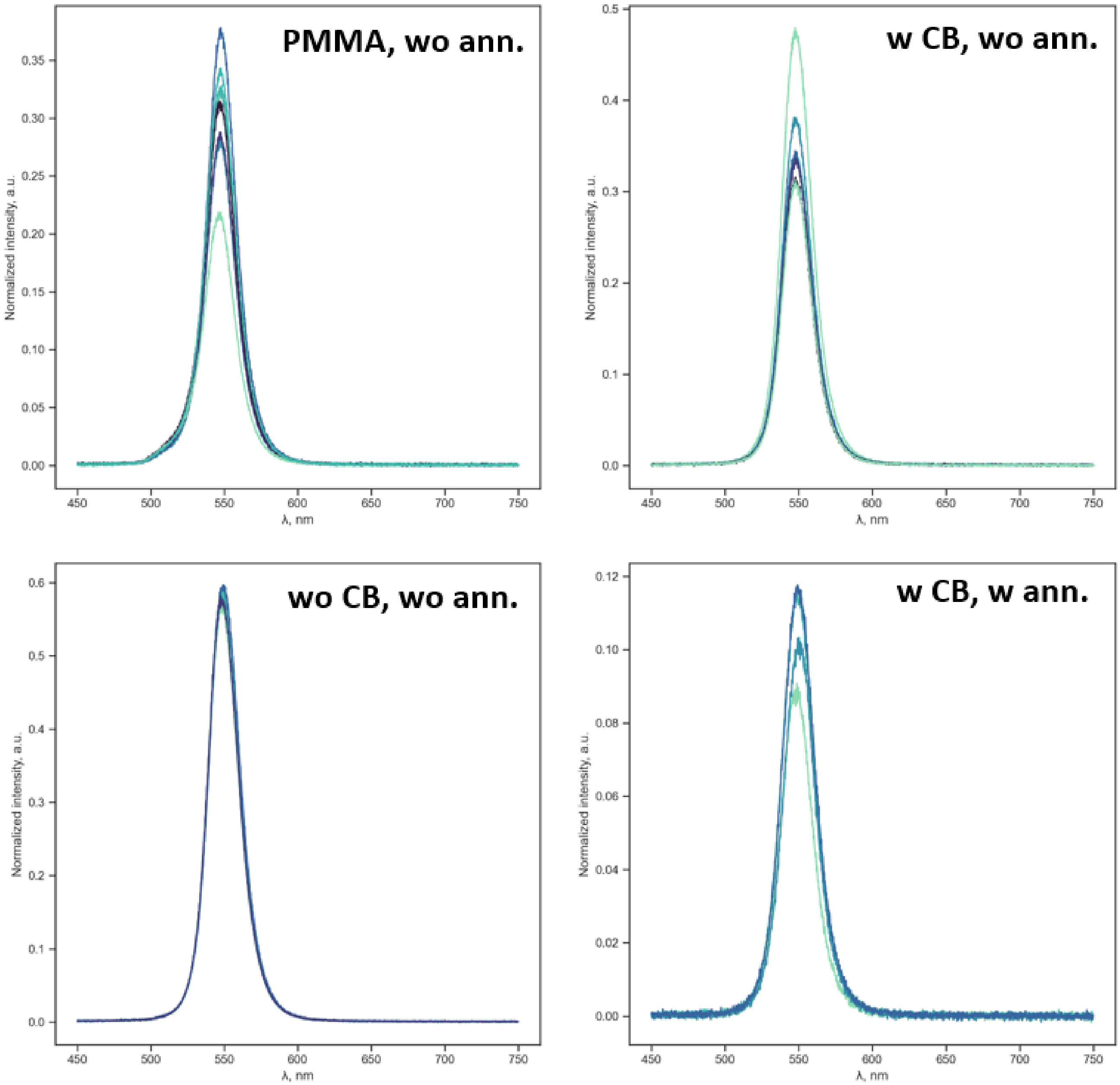

Figure S6. The PL spectra of the film spin-coated from DMSO solution with $FABr{:}PbBr_2$ ratio of 1.5:1 for different processing conditions. Different spectra for each graph correspond to different points on the sample.

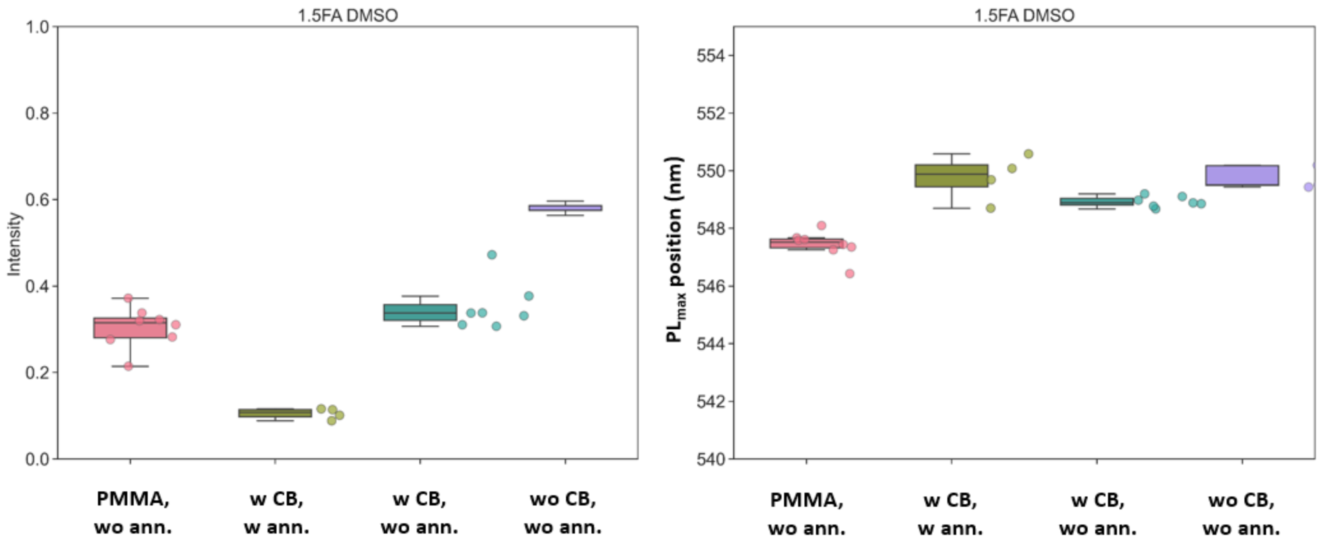

Figure S7. The PL intensity (relative to the standard) and position of PL maximum for different processing conditions (DMSO, $FABr{:}PbBr_2$ = 1.5:1). The statistical spread corresponds to the spectra above.

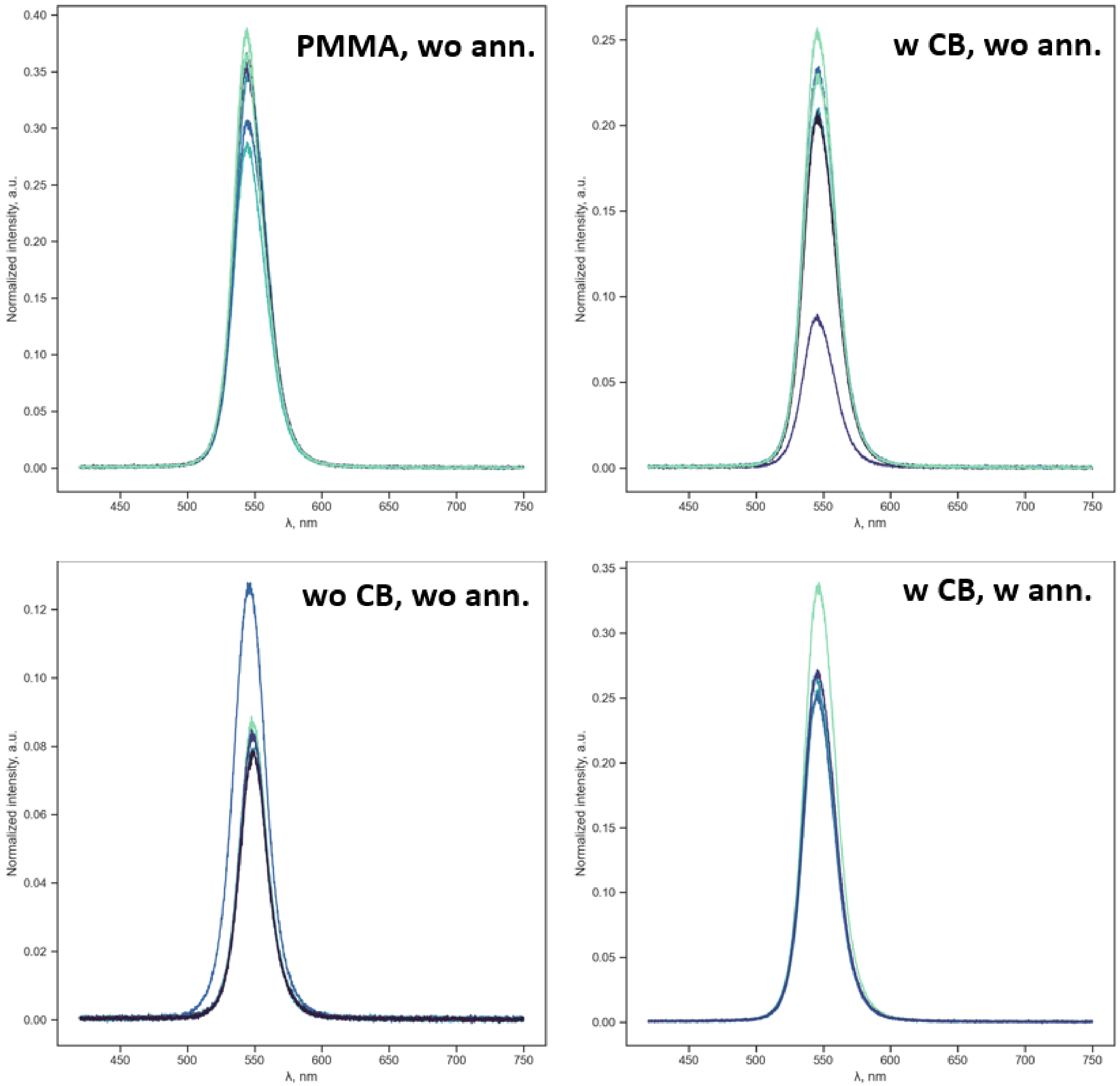

Figure S8. The PL spectra of the film spin-coated from DMF solution with $FABr:PbBr_2$ ratio of 1.5:1 for different processing conditions. Different spectra for each graph correspond to different points on the sample.

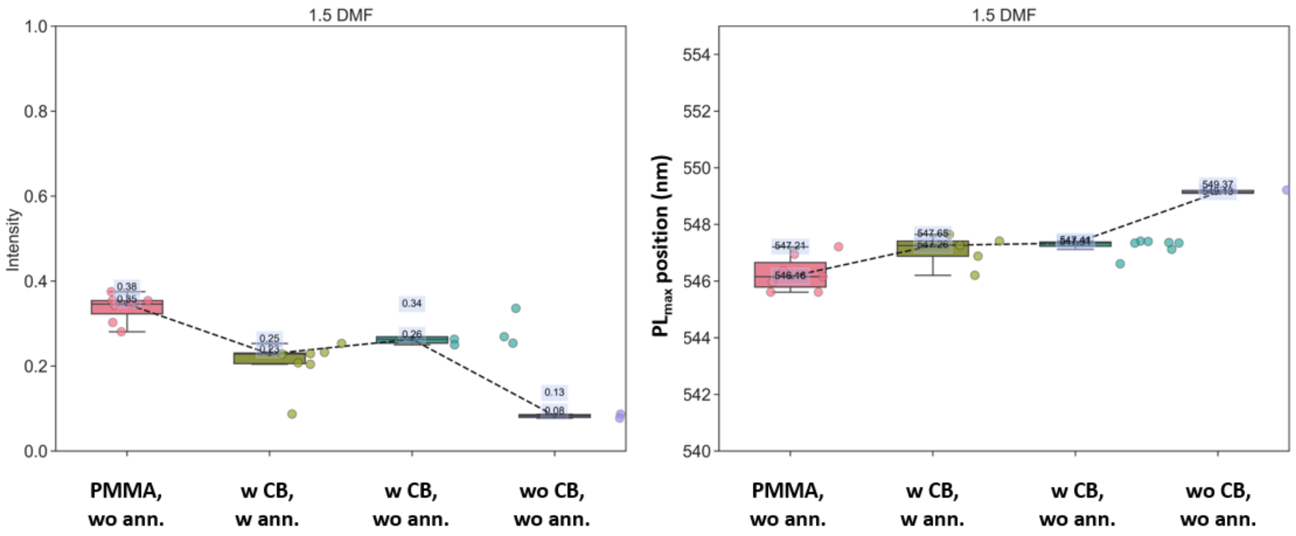

Figure S9. The PL intensity (relative to the standard) and position of PL maximum for different processing conditions (DMF, $FABr:PbBr_2$ = 1.5:1). The statistical spread corresponds to the spectra above.

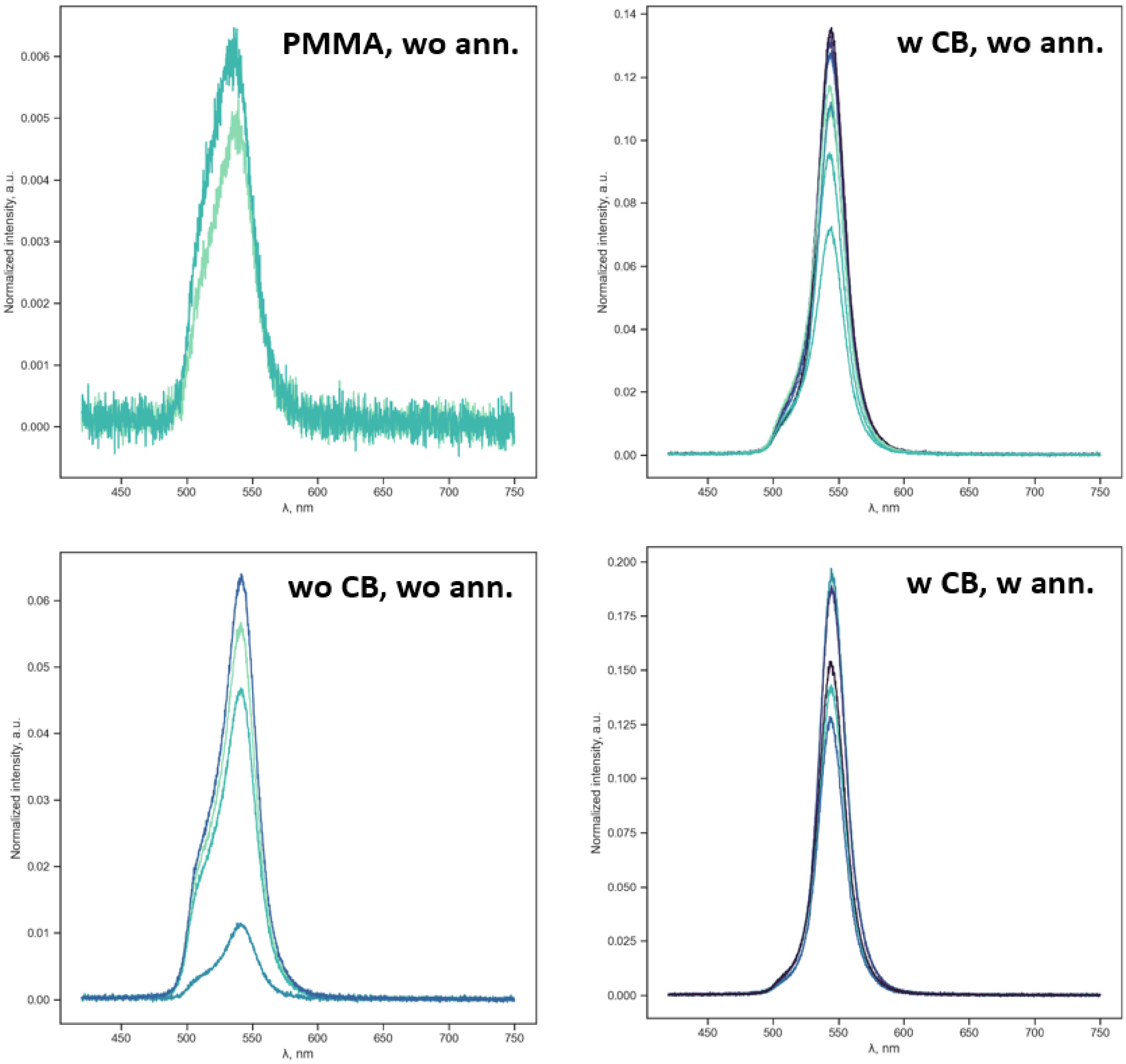

Figure S10. The PL spectra of the film spin-coated from DMSO solution with $FABr:PbBr_2$ ratio of 2:1 for different processing conditions. Different spectra for each graph correspond to different points on the sample.

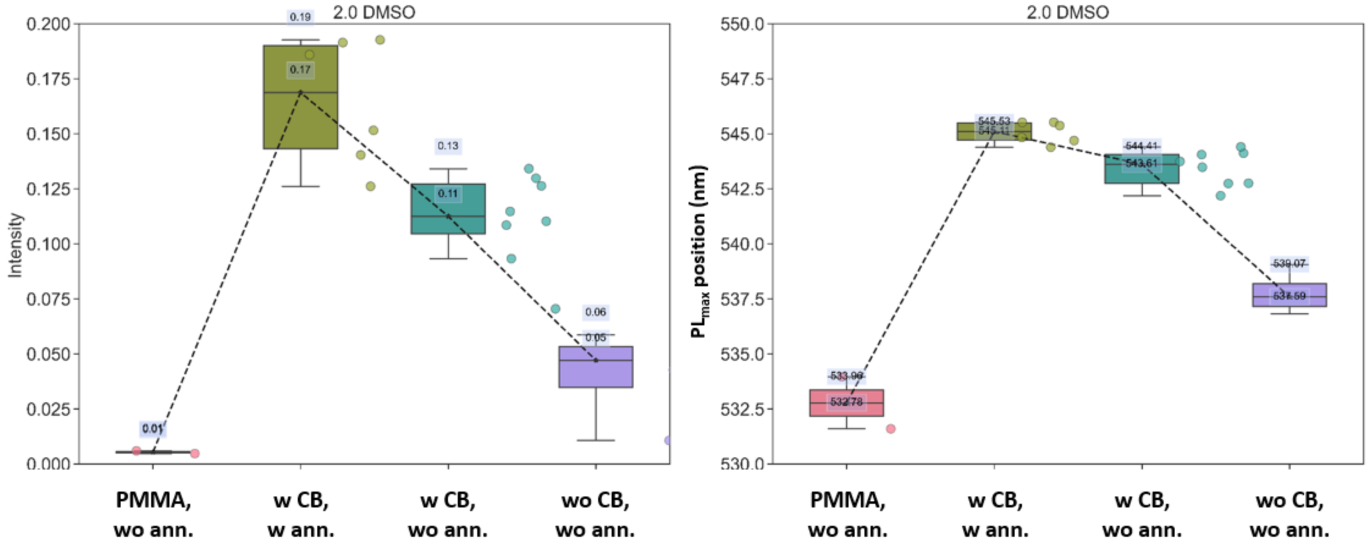

Figure S11. The PL intensity (relative to the standard) and position of PL maximum for different processing conditions (DMSO, $FABr:PbBr_2$ = 2:1). The statistical spread corresponds to the spectra above.

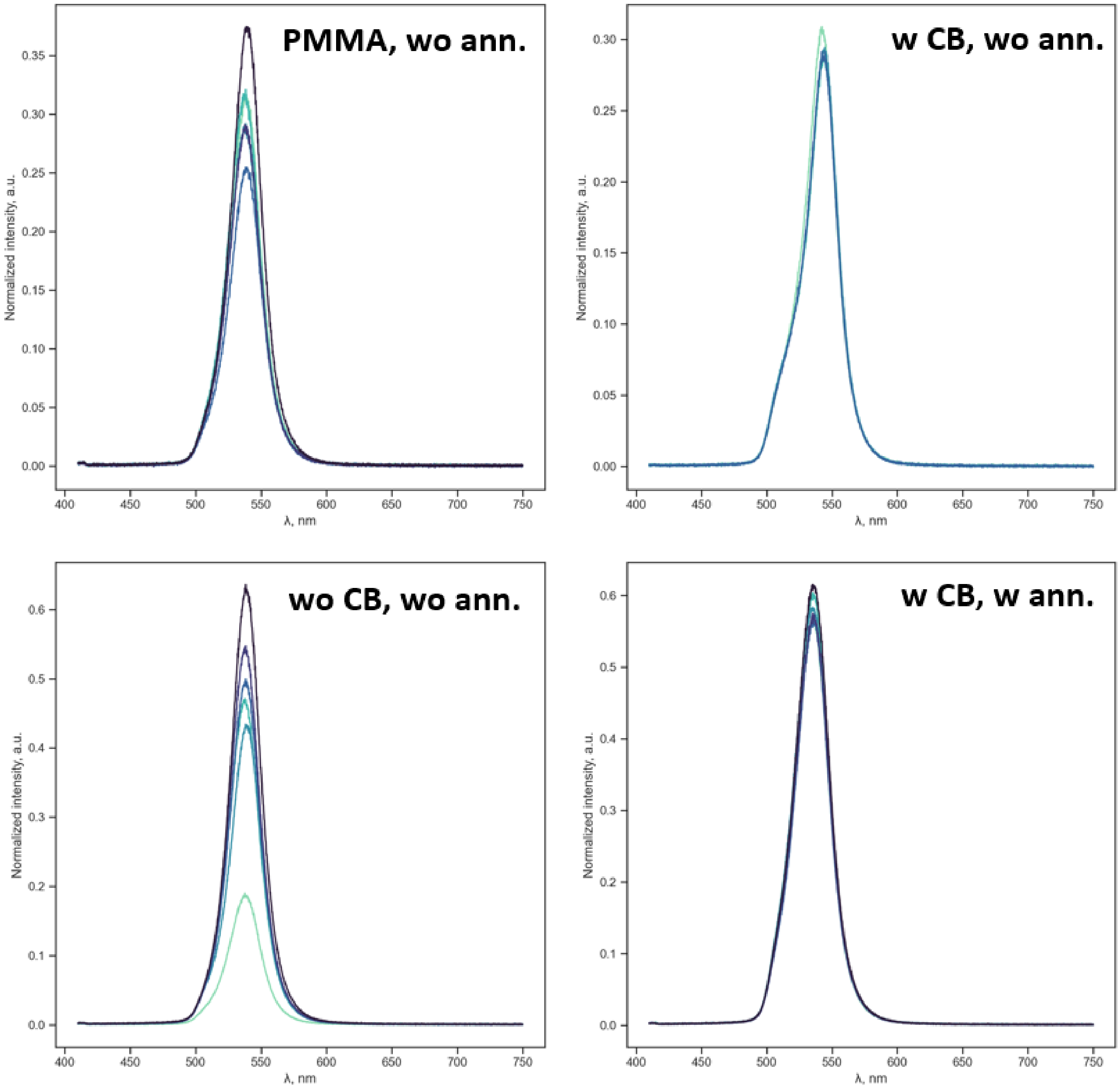

Figure S12. The PL spectra of the film spin-coated from DMF solution with $FABr:PbBr_2$ ratio of 2:1 for different processing conditions. Different spectra for each graph correspond to different points on the sample.

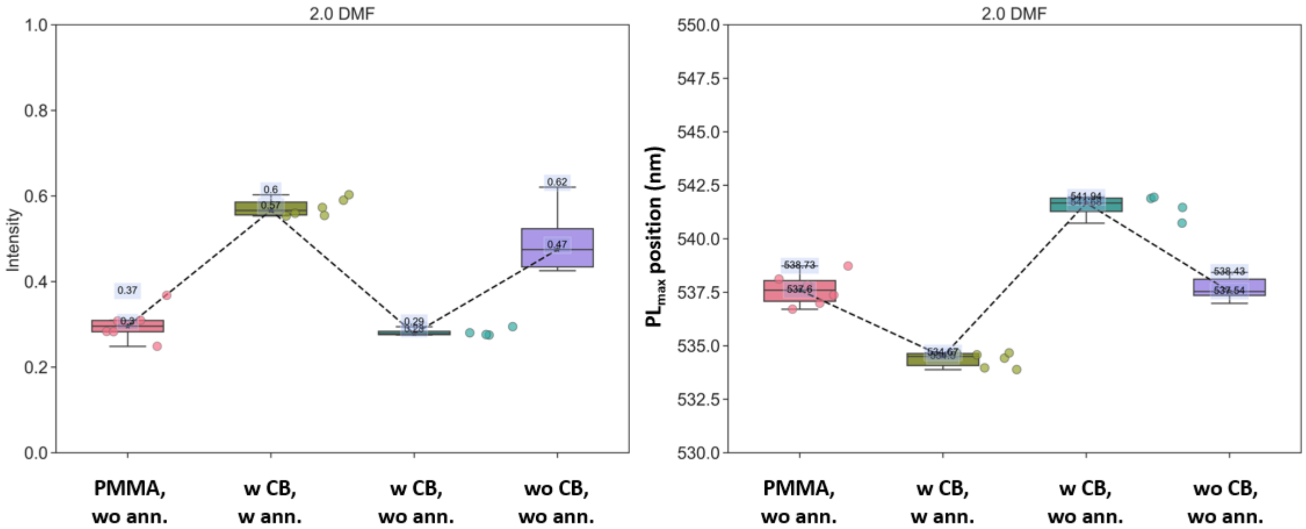

Figure S13. The PL intensity (relative to the standard) and position of PL maximum for different processing conditions (DMF, $FABr:PbBr_2$ = 2:1). The statistical spread corresponds to the spectra above.

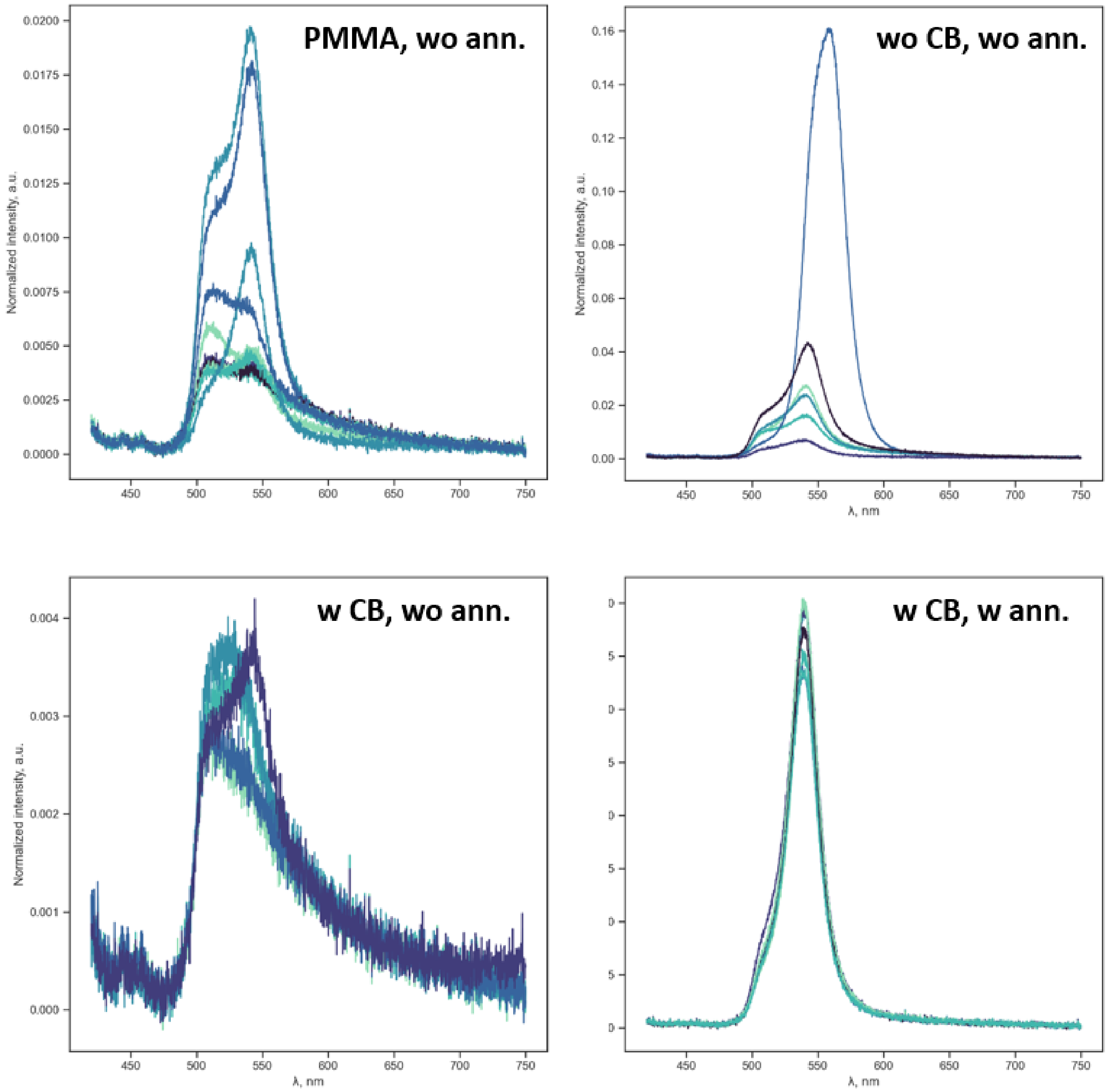

Figure S14. The PL spectra of the film spin-coated from DMSO solution with $FABr:PbBr_2$ ratio of 3:1 for different processing conditions. Different spectra for each graph correspond to different points on the sample.

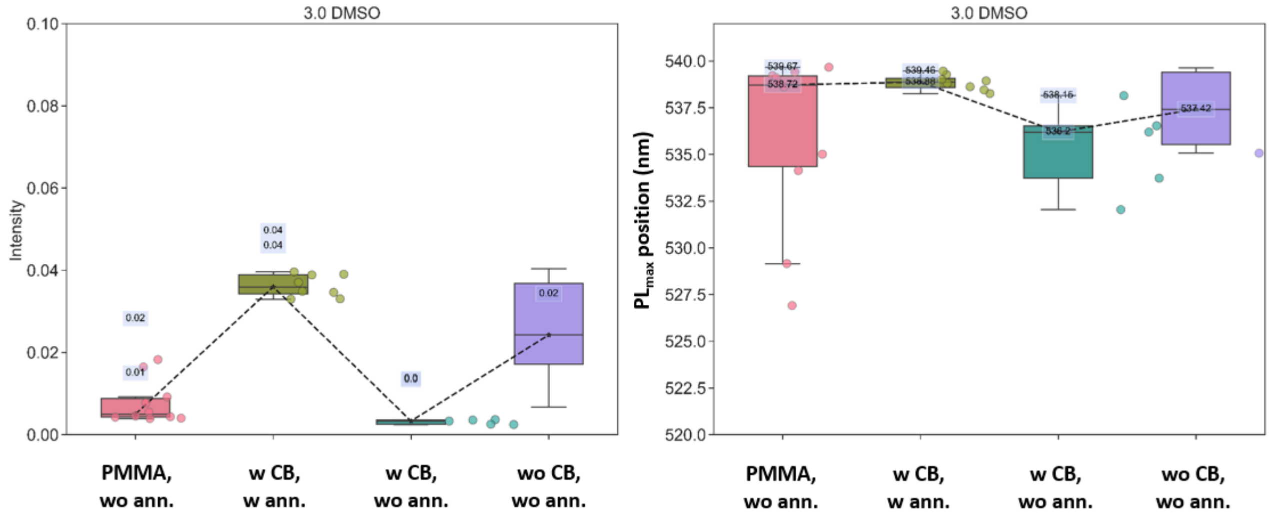

Figure S15. The PL intensity (relative to the standard, multiplied by 50) and position of PL maximum for different processing conditions (DMSO, $FABr:PbBr_2$ = 3:1). The statistical spread corresponds to the spectra above.

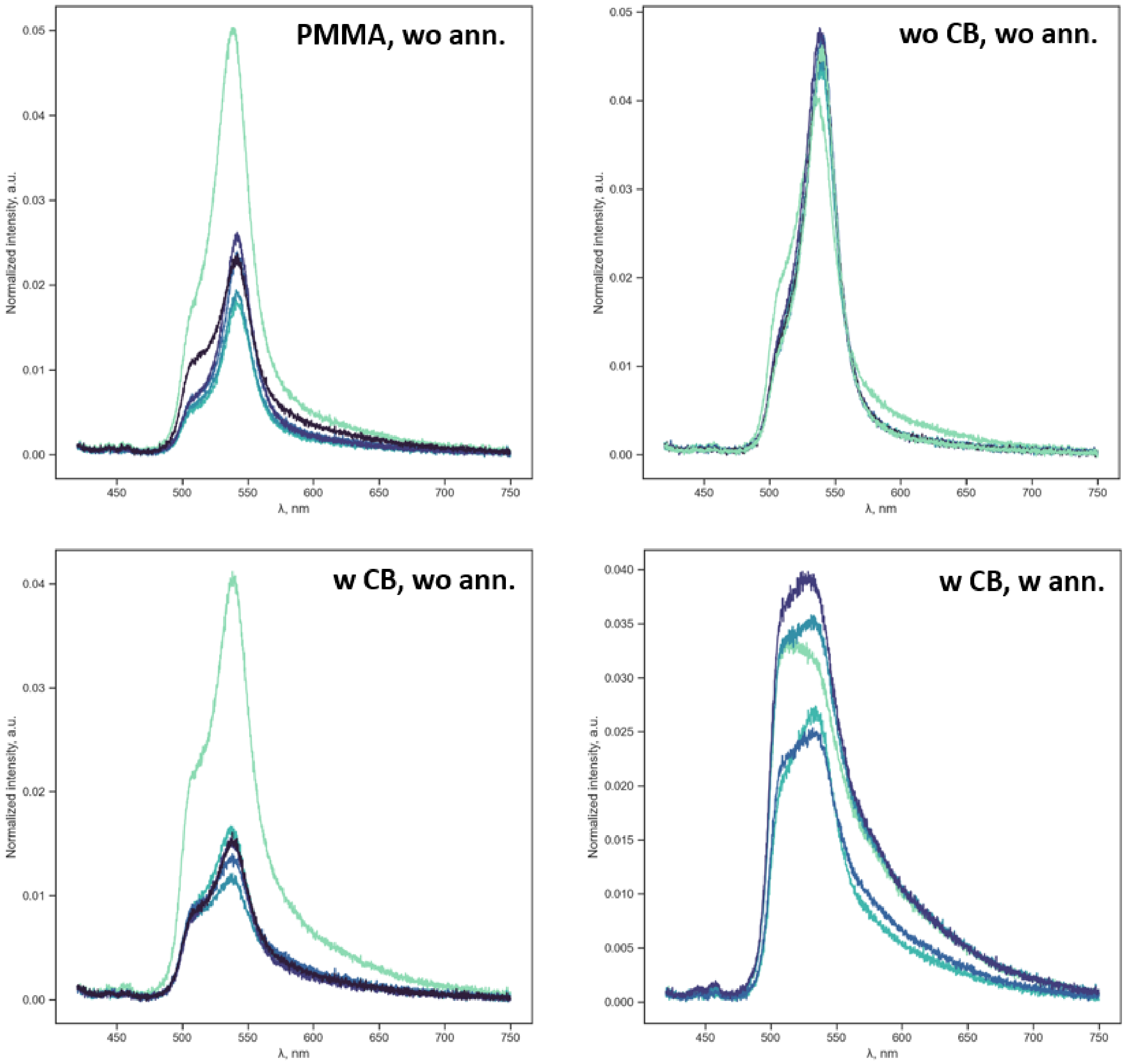

Figure S16. The PL spectra of the film spin-coated from DMF solution with $FABr:PbBr_2$ ratio of 3:1 for different processing conditions. Different spectra for each graph correspond to different points on the sample.

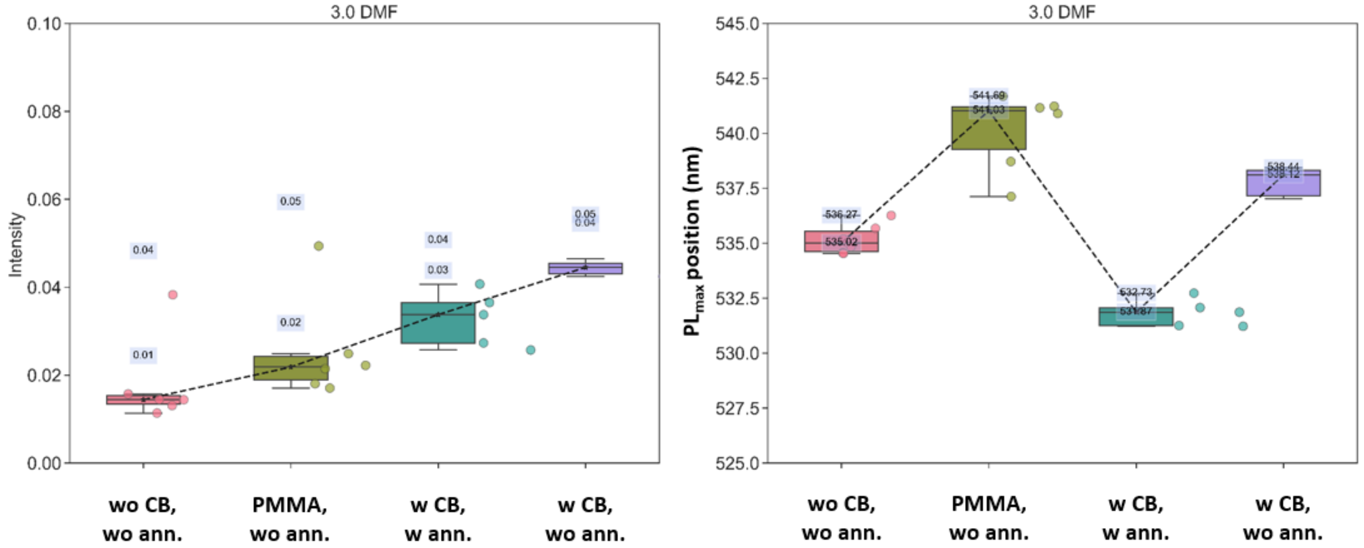

Figure S17. The PL intensity (relative to the standard, multiplied by 50) and position of PL maximum for different processing conditions (DMF, $FABr:PbBr_2$ = 3:1). The statistical spread corresponds to the spectra above.
